# A Behavioral Lifestyle Intervention Enhanced With Multiple-Behavior Self-Monitoring Using Mobile and Connected Tools for Underserved Individuals With Type 2 Diabetes and Comorbid Overweight or Obesity: Pilot Comparative Effectiveness Trial

**DOI:** 10.2196/mhealth.4478

**Published:** 2018-04-10

**Authors:** Jing Wang, Chunyan Cai, Nikhil Padhye, Philip Orlander, Mohammad Zare

**Affiliations:** ^1^ Cizik School of Nursing The University of Texas Health Science Center at Houston Houston, TX United States; ^2^ McGovern Medical School The University of Texas Health Science Center at Houston Houston, TX United States

**Keywords:** self-monitoring, diabetes, obesity, mobile health, behavior change, connected health, patient-generated health data, lifestyle, patient engagement, comparative effectiveness trial

## Abstract

**Background:**

Self-monitoring is a cornerstone of behavioral lifestyle interventions for obesity and type 2 diabetes mellitus. Mobile technology has the potential to improve adherence to self-monitoring and patient outcomes. However, no study has tested the use of a smartphone to facilitate self-monitoring in overweight or obese adults with type 2 diabetes mellitus living in the underserved community.

**Objective:**

The aim of this study was to examine the feasibility of and compare preliminary efficacy of a behavioral lifestyle intervention using smartphone- or paper-based self-monitoring of multiple behaviors on weight loss and glycemic control in a sample of overweight or obese adults with type 2 diabetes mellitus living in underserved communities.

**Methods:**

We conducted a randomized controlled trial to examine the feasibility and preliminary efficacy of a behavioral lifestyle intervention. Overweight or obese patients with type 2 diabetes mellitus were recruited from an underserved minority community health center in Houston, Texas. They were randomly assigned to one of the three groups: (1) behavior intervention with smartphone-based self-monitoring, (2) behavior intervention with paper diary-based self-monitoring, and (3) usual care group. Both the mobile and paper groups received a total of 11 face-to-face group sessions in a 6-month intervention. The mobile group received an Android-based smartphone with 2 apps loaded to help them record their diet, physical activity, weight, and blood glucose, along with a connected glucometer, whereas the paper group used paper diaries for these recordings. Primary outcomes of the study included percentage weight loss and glycated hemoglobin (HbA_1c_) changes over 6 months.

**Results:**

A total of 26 patients were enrolled: 11 in the mobile group, 9 in the paper group, and 6 in the control group. We had 92% (24/26) retention rate at 6 months. The sample is predominantly African Americans with an average age of 56.4 years and body mass index of 38.1. Participants lost an average of 2.73% (mobile group) and 0.13% (paper group) weight at 6 months, whereas the control group had an average 0.49% weight gain. Their HbA_1c_ changed from 8% to 7 % in mobile group, 10% to 9% in paper group, and maintained at 9% for the control group. We found a significant difference on HbA_1c_ at 6 months among the 3 groups (*P*=.01). We did not find statistical group significance on percentage weight loss (*P*=.20) and HbA_1c_ changes (*P*=.44) overtime; however, we found a large effect size of 0.40 for weight loss and a medium effect size of 0.28 for glycemic control.

**Conclusions:**

Delivering a simplified behavioral lifestyle intervention using mobile health–based self-monitoring in an underserved community is feasible and acceptable and shows higher preliminary efficacy, as compared with paper-based self-monitoring. A full-scale randomized controlled trial is needed to confirm the findings in this pilot study.

**Trial Registration:**

ClinicalTrials.gov NCT02858648; https://clinicaltrials.gov/ct2/show/NCT02858648 (Archived by WebCite at http://www.webcitation.org/6ySidjmT7)

## Introduction

More than two-thirds of American adults are overweight or obese [1]. New statistics show that obesity rates are on the rise [2]. Among adults in the United States with diabetes, 80.3% were overweight or obese (body mass index, BMI>25) [3]. Overweight and obesity are major contributors to increased incidence of type 2 diabetes mellitus (T2DM) [4], which is associated with serious comorbid conditions including long-term damage from micro and macrovascular diseases to multiple organs (eg, eyes, kidneys, nerves, heart, and blood vessels) [5]. Strong evidence supports the efficacy of a behavioral lifestyle modification for weight loss, glucose control, and cardiovascular disease (CVD) risk reduction in overweight or obese adults with T2DM [6,7]. The landmark Look AHEAD (Action for Health in Diabetes) trial demonstrated the efficacy of an intensive lifestyle intervention in achieving clinically significant weight loss, glucose control, and CVD risk reduction in overweight or obese adults with T2DM [7]. However, although a recent meta-analysis showed that achieving a weight loss of >5% did demonstrate improvement in metabolic parameters, most studies did not show a weight loss in this range [8]. In particular, few studies provided evidence to support the effectiveness of behavioral lifestyle interventions among underserved populations. A systematic review evaluating behavioral interventions for African Americans with T2DM suggested that clinical trials are needed to tailor interventions to this largely underserved population [9].

Medically underserved populations or patients from medically underserved areas, as defined and designated by the Health Resources and Services Administration [10], are typically older or face barriers to good health and health care based on their income, education, race or ethnicity, or other social and economic factors. Diabetes self-management has been a challenge for all diabetes patients, especially underserved individuals [11,12]. Trief and colleagues found that adherence to diabetes self-management is particularly poor for older minority patients from underserved areas, and adherence is a significant mediator of glycemic control for this population [13]. More innovative and practical strategies are needed to address such disparity and improve glycemic control for underserved T2DM patients.

Self-monitoring of dietary calorie and fat intake and physical activity (PA) was emphasized as a key strategy in the two landmark behavioral lifestyle intervention studies, the Diabetes Prevention Program and the Look AHEAD study [14,15]. In a recent systematic review [16], daily self-monitoring of weight was found to be effective in weight loss without causing negative psychological outcomes. Self-monitoring of carbohydrate intake and self-monitoring of blood glucose (SMBG) are standard practice in diabetes self-management education. However, whether SMBG is effective in the management of T2DM for persons not receiving insulin remains controversial [17]. A systematic review of 30 trials suggested that not using SMBG results to guide corresponding lifestyle behavior changes might have contributed to the inconclusive findings on the effect of SMBG. Thus, we hypothesized that enhancing patients’ problem-solving skills using SMBG results through reflecting, self-monitoring, and regulating diet, activity, and weight could increase the effectiveness of SMBG. Daily self-monitoring of carbohydrate intake, along with self-monitoring of weight and blood glucose, was not part of the included in the two landmark behavioral lifestyle intervention studies [14,15] but holds promise in further improving patient outcomes when used alongside diet and activity self-monitoring.

Although traditional paper diaries were used for self-monitoring in the two landmark behavioral intervention studies, researchers have tested the use of electronic diaries for self-monitoring [18-20] and found these as effective as paper diaries and less burdensome and time consuming. Initially, Burke and colleagues examined the use of personal digital assistants (PDAs) for self-monitoring [18,19] to strengthen the effect of a behavioral weight loss intervention and found PDAs to be a viable alternative that is convenient to use. As technological advances have rendered PDAs obsolete, more recent research tested smartphones to reduce patient burden in self-monitoring and in counting calories using a booklet. Current research comparing the effectiveness of mobile health (mHealth) technology such as PDAs or smartphone apps vs paper diaries to support self-monitoring did not find significant difference on weight loss outcome in several behavioral weight loss trials [21,22]. Moreover, these weight loss trials focused on obese populations only [21,22]; no study compared the two modalities in T2DM patients with comorbid overweight or obesity. Although there are studies testing the use of mHealth tools for diet, PA, and blood glucose self-monitoring as part of a health coaching intervention in T2DM patients [23,24], none of these studies compared the two self-monitoring modalities.

**Figure 1:**
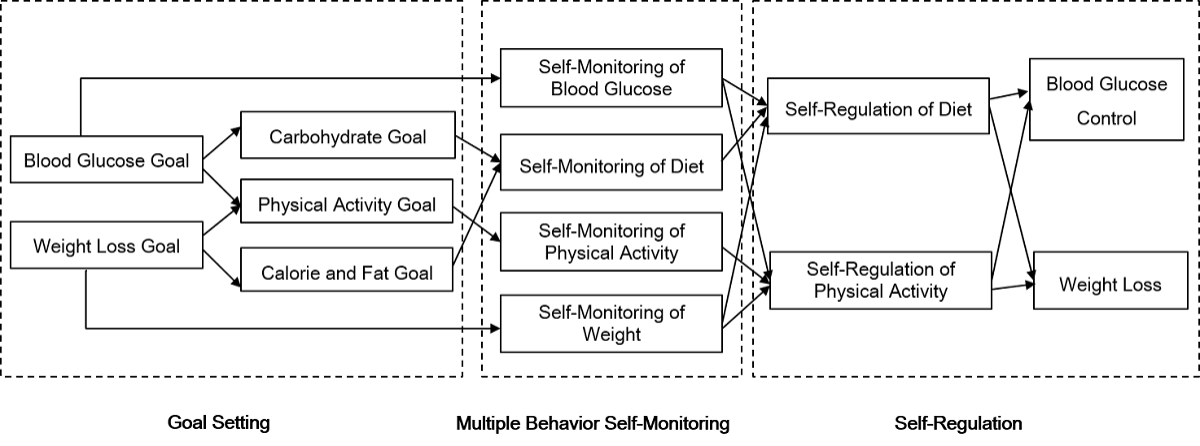
Study model modified from social learning theory and self-regulation theory.

Thus, we propose to fill the scientific gap in testing a behavioral lifestyle intervention for underserved T2DM patients using mHealth tools to enhance multiple-behavior self-monitoring of diet, PA, weight, and blood glucose in a pilot comparative effectiveness trial. On the basis of self-regulation theory, we hypothesized that monitoring multiple behaviors (ie, calorie and fat consumption, exercise, and carbohydrate intake) and associated health outcomes (ie, weight and blood glucose levels) simultaneously can result in behavior change through better self-awareness of how eating and exercise play a role in both weight and glycemic control (Figure 1). In this study, we sought to assess the feasibility of this mHealth-enhanced intervention and compare its preliminary efficacy with that of paper-based multiple-behavior monitoring and standard diabetes care and education in improving glycemic outcomes among overweight or obese adults with T2DM living in underserved communities.

## Methods

### Study Design

We conducted a three-group pilot randomized controlled clinical trial comparing the efficacy of a behavioral lifestyle intervention modified for underserved populations using either (1) mobile or (2) paper-based tools for self-monitoring of diet, PA, weight, and blood glucose and (3) usual diabetes care and education on glycemic control and weight loss at 3 and 6 months. We used a mixed-method design with quantitative measures to evaluate the feasibility and preliminary efficacy of the intervention and conducted focus groups to assess participants’ acceptability of the intervention. We are reporting the quantitative study findings in this paper. The study is approved by the institutional review board at the University of Texas Health Science Center at Houston. Consolidated Standards of Reporting Trials (CONSORT) of Electronic and Mobile HEalth Applications and onLine TeleHealth was used to guide the reporting of this study; a checklist was uploaded as Multimedia Appendix 1 in this paper [25].

### Sample and Sample Size

Participant inclusion and exclusion criteria are shown in Textboxes 1 and 2.

We used the age criteria from the Look AHEAD clinical trial and limited the exclusion criteria to include a general clinical population with a goal to be pragmatic in the nature of this study. To obtain a more representative sample of the underserved population, we included patients with amputations as long as they were able to perform regular activity such as walking. Due to the nature of this study being a pilot and feasibility study, we aimed to recruit 30 patients with 10 patients in each group.

### Setting

Participants were recruited from an American Diabetes Association certified diabetes education program located in a community health center primarily serving uninsured or underinsured individuals living in Harris County, Texas. Flyers were distributed to the patients attending diabetes self-management education classes by their diabetes educators.

### Enrollment and Randomization

If patients showed interest in this study and approached the study team for more details, they were screened for eligibility based on the inclusion and exclusion criteria; their diagnosis of T2DM was confirmed via chart review. Informed consent was obtained from all eligible study participants before enrollment. The study participants were aware of the differences among the three randomization groups during the consenting process. After consent and enrollment, we assigned each patient with a study ID number. The study statistician generated a randomization sheet with the group assignment for each study ID. Study participants were then randomly assigned to one the three study groups based on the randomization sheet.

### Intervention

#### Intervention Overview

To increase study replicability, key differences among the three randomization groups and the behavioral lifestyle intervention in the landmark Look AHEAD trial (intervention materials are publicly available at look ahead trial website) are presented in Table 1.

**Inclusion criteria.**

Individuals were included if they

- had a diagnosis of type 2 diabetes mellitus (T2DM) for at least 6 months by self-report and later confirmed in the electronic health records
- were overweight or obese (body mass index, BMI>25)
- were aged 21 to 75 years
- were able to read and write in English
- had completed or were about to complete the basic diabetes self-management education offered at the recruitment site

**Exclusion criteria.**

Individuals were excluded if they

- had a history of severe psychiatric disorders (eg, bipolar disorder or schizophrenia)
- were unable to perform regular activity
- were currently or planned to be pregnant or nursing in the next 6 months
- had a planned vacation in the next 6 months
- had previously participated in an intensive behavioral lifestyle intervention
- had substance abuse in the past year

**Table 1:** Comparison of key intervention components among three randomization groups and standard behavioral lifestyle intervention used in the landmark Look AHEAD (Action for Health in Diabetes) trial. SMBG: self-monitoring of blood glucose.

Intervention components		Look AHEAD (Action for Health in Diabetes)	Paper group	Mobile group	Usual care and education
**Self-monitoring**					
	Self-monitoring of diet	Paper diaries given to monitor meals, calories, fat goals	In addition to Look AHEAD protocol, add a focus on self-monitoring of carb intake, SMBG, and self-monitoring of weight	Use smartphone app with connected glucometer to monitor the same parameters as the paper group	No, diabetes educator may do one dietary recall during an education visit or give general recommendations to carb counting
	Self-monitoring of physical activity	Paper diaries given to monitor exercise minutes, calories burned	Same as Look AHEAD group	Smartphone app	No
	Daily self-monitoring of weight	Not part of the intervention	Yes, a weight scale, and place in a paper diary to document	Provide a wireless weight scale and its companion smartphone app for daily weight monitoring	No
	Self-monitoring of blood glucose	Not part of the intervention	Recommend every other day at the recruiting center, free glucometer and strips once every other day, our study will supplement strips for daily SMBG	Provide wireless glucometer and its companion smartphone app and strips for daily SMBG	Recommend every other day at the recruiting center, free glucometer and strips once every other day, our study will supplement strips for daily SMBG
Behavioral intervention sessions		Month 1-6, weekly sessions (3 group + 1 individual)	11 group sessions + 1 individual session in the first 6 months	Same as paper group	No
Usual care and diabetes education			Same as usual care and education group	Same as usual care and education group	3 group classes and follow up classes as needed with diabetes educators, physician visit about every 6 months depending on condition
Meal replacement		Yes	No	No	Not recommended at the recruitment site

##### Usual Diabetes Care and Education Group

Participants in the control group received usual care and diabetes education from their primary care physicians and diabetes educators. The recruiting community health centers offer a diabetes education program for all diabetes patients. The diabetes education program consists of individual visits or a series of two interactive group classes taught by registered dietitians or nurses who are certified diabetes educators. The topics covered in the sessions are as follows: SMBG skills, carbohydrate counting, healthy eating and exercise, and the risk and management of hyperglycemic and hypoglycemic situations. Patients are not typically asked to self-monitor diet, activity, and weight on a daily basis in diabetes education. During diabetes education, patients typically set one to three behavioral goals centered on nutrition, PA, risk prevention, SBMG, or medication.

##### Group and Individual Behavioral Lifestyle Intervention Sessions for Both the Mobile and Paper Groups

In addition to receiving usual diabetes care and education at the recruiting community center, both the mobile and paper groups received a standard behavioral lifestyle intervention comprising 11 group sessions—weekly for month 1, biweekly for months 2 and 3, and monthly for months 4 to 6—and an individual session after month 3. The group sessions were held at the recruiting community health center and included a grocery shopping trip. Pedometers, weight scales, and food scales were distributed in the sessions. The topics for the 11 sessions were as follows: (1) Welcome to the Program; (2) Be a Fat and Calorie Detective; (3) Healthy Eating; (4) Grocery Shopping and Cooking; (5) Move Those Muscles, Jump Start Your Activity Plan; (6) Tip the Calorie Balance, Take Charge of What’s Around You; (7) Problem Solving, Stress, and Time Management; (8) Four Keys to Healthy Eating Out, Make Social Cues Work for You; (9) Slippery Slope of Lifestyle Change, Ways to Stay Motivated; (10) Prepare for Long-Term Self-Management, More Volume or Fewer Calories; and (11) Balance Your Thoughts, Strengthen Your Exercise Program. Each session took approximately 1 to 2 hours.

Two lifestyle counselors were trained using publicly available materials and a digital optical disc and printed training materials from the Group Lifestyle Balance (GLB) program and the Look AHEAD intervention. On the basis of GLB and Look AHEAD intervention principles, a standard behavioral intervention program typically includes group sessions focused on the following behavioral strategies: (1) goal setting, (2) feedback, (3) portion control, (4) cooking class, (5) field trip, (6) social support, (7) incentives, (8) problem solving, (9) relapse prevention, and (10) self-monitoring. All of these strategies in the original 12 core sessions and four transition sessions in the first 6 months of the GLB program were integrated and delivered in the 11 group sessions. An individual intervention was added ad hoc to evaluate individualized goals and behavior change plans; review individual weight loss goals, current weight, and diaries; how to tip the calories; and develop specific diet and PA goals to reach weight loss goal.

To adapt the intervention for the underserved population, all intervention materials were modified to be at 9th grade reading level. Intervention sessions were delivered at the recruiting community health center that is close to most of the participants’ homes. The grocery shopping trip was also conducted in the neighborhood where the participants typically shop.

##### Multiple-Behavior Self-Monitoring Intervention for the Mobile and Paper Groups

Participants received training on how to self-monitor their diet and exercise habits, weight, and blood glucose in the first two sessions. Specifically, both groups were instructed to record their exercise activities (minutes and type of activity) and specify the foods they ate; the amount eaten; the number of calories, fat grams, and carbohydrates; their weight; and their blood glucose using a paper diary or an electronic diary depending on their group randomization.

Mobile group: for those who did not have a smartphone, we provided a smartphone for use over 6 months. None of the participants assigned to this group owned a smartphone, so all study participants in the group were given a smartphone with two apps downloaded by the study team. The participants used the LoseIt! (FitNow, Inc, Boston, Massachusetts) smartphone app for self-monitoring of diet, PA, and weight and the Diabetes Connect app (PHRQL Inc, Pittsburgh, Pennsylvania) connected with MyGlucoHealth, a Bluetooth-enabled glucometer (Entra Health Systems LLC, San Diego, California). There were no prompts or reminders embedded in these apps; however, we discussed self-monitoring results and encouraged participants to share experience using them during the 11 face-to-face group sessions.

Paper group: we provided CalorieKing food and exercise journals to study participants to write down their daily dietary intake and exercise. We instructed them to record their weight and blood glucose levels on the same pages, with the goal of helping them make connections between their diet, PA, weight, and glucose outcomes. Free stand-alone glucometers were provided to all patients at the recruitment sites. A CalorieKing counter, calculator, food scale, and food measuring set was provided to each participant in the paper group to measure their food portions; look up calorie, fat, and carbohydrate content; and calculate the total numbers for dietary self-monitoring.

### Treatment Fidelity

A checklist was developed and used for each group and individual session to track the content delivered. The principal investigator (PI) attended at least 80% of the group sessions for both paper and mobile groups to ensure treatment fidelity. Training of the two lifestyle counselors (their backgrounds were in public health and kinesiology) occurred 4 months before the study. Mock sessions were conducted on weekly meetings where lifestyle counselors developed PowerPoint slides, delivered mock intervention sessions, and reviewed the checklist for each session.

#### Missed Sessions

Individual or group make-up sessions were scheduled for those who had to miss any group or individual sessions.

### Measures

#### Feasibility: Retention, Group Session Attendance, and Adherence

Study feasibility was evaluated using retention rates at 3 and 6 months, group session attendance rates, and adherence to self-monitoring for both intervention groups. Participants in both intervention groups were asked to assess the acceptability of the 6-month behavioral intervention. Focus groups were conducted at the end of the intervention to learn about participants’ experiences and satisfaction with the intervention.

#### Preliminary Efficacy

All of the outcome measures were administered at baseline, 3 months, and 6 months. The study was completed in 2015. Physical measurements and a blood samples were obtained at the study sites.

#### Primary Outcome Measure-Glycemic Control

Glycemic control was determined by glycated hemoglobin (HbA_1c_) levels. Patients were asked to fast for at least 8 hours before the scheduled data collection visits for venipuncture. A healthy breakfast including fresh fruits and breakfast bars was offered after blood draws. Blood samples were then transferred to a biological laboratory for analysis.

#### Secondary Outcome Measure-Weight

We used a Tanita scale and body fat analyzer (Tanita Corporation of America Inc, Illinois, United States) to measure weight and body composition while subjects wore light clothing and stood erect with their bare feet on the scale’s footpads.

#### Sociodemographic and General Health Information

Participants’ age, gender, ethnicity, race, marital status, education level, employment status, weight, and diabetes history were collected in a sociodemographic questionnaire. Details about their personal health and medical history (eg, comorbid conditions) were collected in a general health history form.

### Data Management

The recruitment, feasibility, and tracking forms were collected and stored in the PI’s office at the Cizik School of Nursing at The University of Texas Health Science Center at Houston for data processing. Oracle (version 9i, Oracle Corporation, Redwood Shores, California) was used for data management. Form design, data entry, and data verification were performed in TeleForm (version 10.0, Verity Inc, Sunnyvale, California) for automated data entry or verification. All forms were precoded to minimize coding errors. During data collection, forms were screened upon receipt for completeness of response. Once verified, data were exported to the Oracle database for further data processing before being exported to SAS (SAS Institute) for data analysis.

### Statistical Analysis

Intention-to-treat (ITT) analyses were performed on the primary and secondary outcomes. Descriptive statistics (frequency and percentage for categorical variables and mean and SD or median and interquartile range for continuous variables) were reported for retention at 6 month of the intervention, attending group sessions, and adherent to the multiple-behavior self-monitoring. For continuous variables with skewed distribution (eg, retention rates and group session attendance rates), nonparametric Mann-Whitney *U* tests were conducted for comparison between the mobile and paper groups. For the primary outcome, the percentages of weight change over time were compared by Kruskal-Wallis test, and the percentages of HbA_1c_ change over time were compared by analysis of variance. Sensitivity analyses were conducted using last observation carried forward (LOCF) method to impute the missing data for participants who withdrew or were lost to follow-up.

## Results

### Sociodemographic Characteristics

Demographic characteristics of the sample by randomization group are presented in Table 2. The average age of the participants was 56.4 years, and the average years of education were 12.15 years (SD 1.22). A total of 62% (16/26) of the sample were female, and 69% (18/26) were African Americans. The BMI ranged from 27.4 to 51.1, with average of 38.1 at baseline. The majority of the sample had no health insurance or received only Medicare or county-assisted insurance in Harris County, Texas. All of the study participants were uninsured or underinsured. The household income for all study participants was below US $30,000, and 92% (24/26) had a household income lower than US $20,000. Age (*P*=.04) and gender (*P*=.07) differed significantly among the three randomization groups, but no statistically significant differences were found among other demographic variables.

### Feasibility

#### Retention and Group Session Attendance

**Figure 2:**
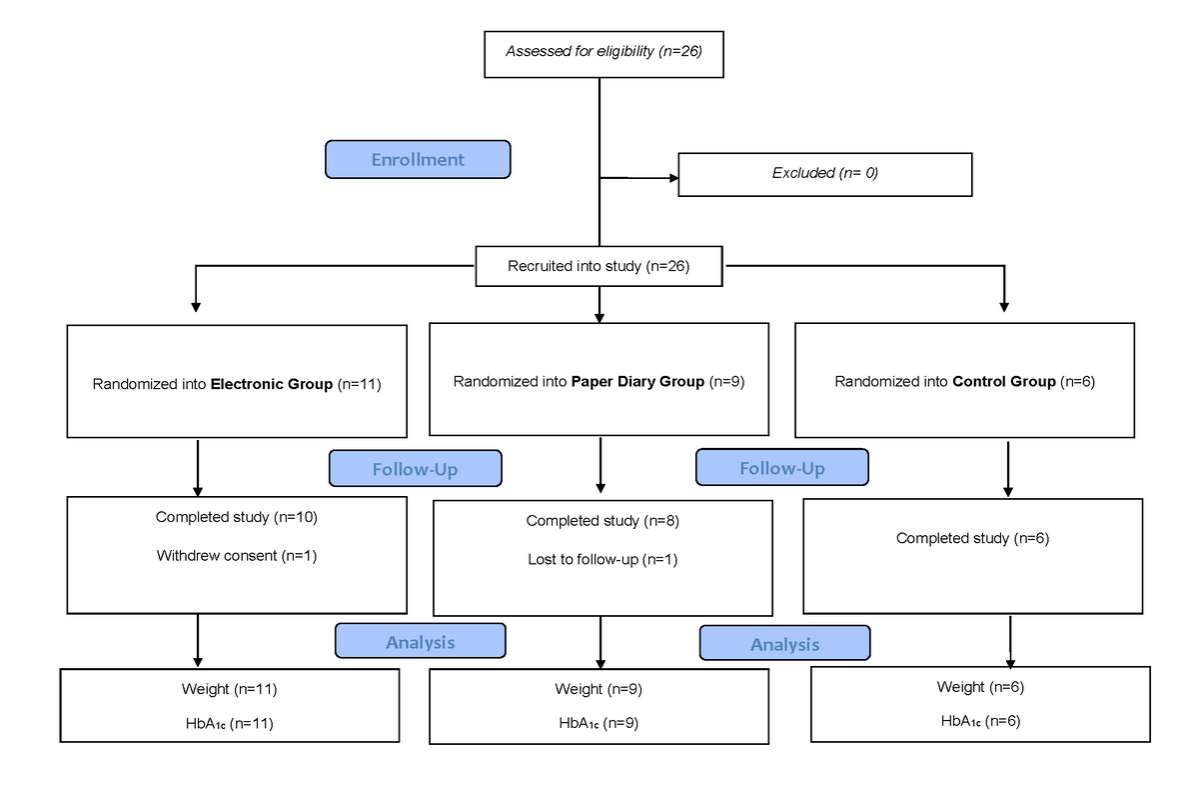
Consolidated Standards of Reporting Trials (CONSORT) diagram. HbA_1c_: glycated hemoglobin.

One person dropped out of the study before the intervention started because of a schedule conflict for group sessions. The retention rate at 3 months was 96% (25/26) and 92% (24/26) at 6 months. Retention rates were not significantly different in the three randomization groups (*P*>.05). The CONSORT diagram depicting patient retention is in Figure 2.

The median rate of session attendance at the 11 group sessions was 100% (range from 54.5%-100%) for the mobile group and 81.8% (range from 27.3%-100%) for the paper group. The nonparametric Mann-Whitney *U* test showed a statistically significant difference in group session attendance between the mobile and paper groups (*P*=.01).

#### Patient Engagement and Adherence to Self-Monitoring

In the mobile group, the median percentage of days with at least one self-monitoring entry for diet, PA, weight, and glucose was 96.6%, 37.3%, 49.7%, and 72.7%, respectively, whereas the corresponding median adherence rates for the paper group were 8.1%, 1.2%, 2.5%, and 2.5%. Nonparametric Mann-Whitney *U* tests showed that there were significant differences between the mobile and paper group in all four self-monitoring variables (*P* ≤.001 for diet and PA, *P*=.007 for weight, and *P*=.003 for glucose).

**Table 2:** Demographic characteristics by three groups.

Variables		Mobile group (N=11)	Paper group (N=9)	Control group (N=6)
Age (years), mean (SD)		58.8 (5.9)	56.1 (5.4)	49.2 (10.2)
Female, n (%)		9 (82)	5 (56)	1 (20)
Body mass index, mean (SD)		38.9 (9)	40.1 (7.0)	33.7 (2.7)
**Ethnicity, n (%)**				
	Not Hispanic	9 (73)	7 (67)	4 (67)
	Hispanic	2 (18)	2 (22)	2 (33)
**Race, n (%)**				
	White	3 (27)	2 (22.2)	2 (3)
	Black	7 (64)	6 (67)	4 (80)
	American Indian	0 (0)	1 (11)	0 (0)
	Asian	1 (11	0 (0)	0 (0)
**Employment status, n (%)**				
	Full time	2 (18)	1 (11)	0 (0)
	Part time	0 (0)	2 (22)	2 (33)
	Laid off	2 (18)	0 (0)	0 (0)
	Retired	3 (27)	1 (11)	1 (17)
	Disabled or unable to work	2 (18)	5 (56)	1 (17)
	Full time homemaker	2 (18)	0 (0)	1 (17)
	Student	0 (0)	0 (0)	1 (17)
**Insurance coverage, yes, n (%)**		7 (64)	4 (44)	1 (17)
**Insurance type, n (%)**				
	Medicare	5 (71)	3 (60)	0 (0)
	Gold Card, Harris County	2 (29)	2 (40)	2 (100)
**Income (USD), n (%)**				
	Under $10,000	1 (10)	3 (33)	2 (33)
	$10,000-$13,000	5 (50)	0 (0)	1 (17)
	$13,000-$20,000	3 (30)	6 (67)	2 (33)
	$20,00-$30000	1 (10)	0 (0)	1 (17)
Years of education, mean (SD)		12.3 (1.0)	11.8 (1.5)	12.5 (1.2)

**Figure 3:**
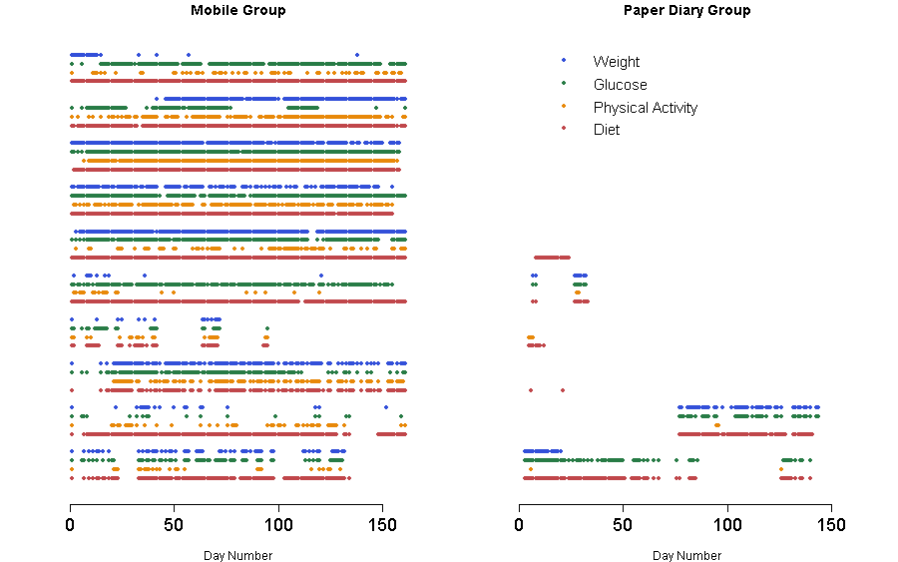
Adherence to self-monitoring of multiple behaviors in the intervention groups.

Figure 3 depicts the frequency of days on which each of the four self-monitoring variables was reported for each of the 10 study participants in the mobile group and for each of the 6 study participants in the paper diary group.

### Evaluation of Intervention on Outcomes (Preliminary Efficacy)

Descriptive findings on HbA_1c_ and weight outcomes at each study data collection time point (baseline, 3 months, and 6 months) are summarized in Table 3. At baseline, there were no statistical significant differences on HbA_1c_ among the three randomization groups; at 6 months, a statistical significant difference on HbA_1c_ was found among the three groups, with mobile group having an average HbA_1c_ level <7%, whereas the paper group and control group had an average HbA_1c_ level around 9%. Results from the ITT analysis on the primary outcome of HbA_1c_ showed that there were no statistical significant group differences on HbA_1c_ level change over 6 months (*P*=.44); however, a medium effect size of Cohen *d*=0.28 was detected for HbA_1c_ changes. At 6 months, participants in the mobile group had an average weight loss of 1.8%, whereas the paper group had an average of 4% weight gain, and the control group had an average of 1.6% weight gain. There were no statistical significant differences among the three groups on weight changes over time (*P*=.20). A medium effect size of Cohen *d*=0.40 was found for changes on weight outcomes over time. Sensitivity analysis using LOCF for imputations did not show any statistically significant differences on the HbA_1c_ and weight outcomes.

**Table 3:** Descriptive values for weight and glycated hemoglobin (HbA_1c_) levels at each visit by group. Q1: 25^th^ percentile; Q3: 75^th^ percentile.

Variables		Mobile group (N=11)	Paper group (N=9)	Control group (N=6)	*P* value
**HbA_1c_, mean (SD)**					
	Baseline	8.4 (2.3)	10.4 (2.4)	8.9 (2.4)	.20
	3 months	7.3 (1.1)	8.5 (1.4)	8.5 (1.7)	.13
	6 months	6.9 (1.0)	9.1 (1.8)	8.9 (1.6)	.01
**Weight, median (Q1, Q3)**					
	Baseline	233.6 (179.8, 295.4)	243.6 (222.2, 321.8)	201.2 (195.8, 213.8)	.48^a^
Percentage weight change at 3 months, median (Q1, Q3)		0.5 (−2.9, 2.2)	−1.0 (−1.6, −0.1)	2.1 (0.1, 4.2)	.16^a^
Percentage weight change at 6 months, median (Q1, Q3)		−1.8 (−4.2, −0.3)	0.4 (−2.3, 1.5)	1.6 (−4.1, 3.8)	.16^a^

^a^Denotes *P* values obtained from Kruskal-Wallis test; other *P* values were obtained from analysis of variance.

## Discussion

### Principal Findings

To our knowledge, this pilot study is the first to report the feasibility and acceptability of using mobile and connected tools to enhance an evidence-based behavioral lifestyle intervention for the underserved community. We compared the efficacy of standard diabetes care and education with behavioral lifestyle interventions enhanced with either using smartphone apps and a Bluetooth-connected glucometer for self-monitoring of multiple behaviors or paper diaries on improving glycemic outcomes among overweight or obese adults with T2DM living in underserved communities. The feasibility and acceptability of the study were demonstrated by the high retention rates at 3 and 6 months and high rates of patient engagement in using the mobile apps. In fact, our retention rates of 96% at 3 months and 92% at 6 months were higher than those reported in most of the previous behavioral lifestyle interventions mediated by technology in obesity and T2DM [26,27], including those in medically underserved communities [28].

The comparative findings revealed the mobile group participants had higher group session attendance and higher patient engagement and adherence to self-monitoring of multiple behaviors than the paper group, which was consistent with previous studies reporting higher adherence to self-monitoring rates using electronic diaries compared with paper diaries among overweight or obese populations [29,30]. As compared with the diabetes population, a study planning to use a mobile app to support patient self-management did not recruit enough patients [31]. We recruited our 27 patients in less than 1 month, and those who participated in the mobile group had high adherence rates to all components of the intervention over 6 months. This may suggest that a mobile app alone does not interest patients as much as a hybrid of face-to-face sessions using mobile apps to support self-monitoring.

Although the previous literature comparing electronic diaries and paper diaries for self-monitoring of diet and PA did not reveal significant differences in weight loss outcomes [21,32], our study not only showed significantly better adherence to self-monitoring in the mobile group but also a trend for greater weight loss and glycemic control with medium effect sizes in the mobile group. Furthermore, the mobile group had significantly lower HbA_1c_ levels at 6 months than the paper group. A meta-analysis of lifestyle weight loss interventions in overweight and obese adults with T2DM revealed that the majority of the trials did find <5% weight loss; however, they did not reveal significant beneficial effects on glycemic control [33]. The self-monitoring intervention in these lifestyle weight loss interventions focused on self-monitoring of diet and PA only, with a few of them adding self-monitoring of weight, whereas our study used a holistic approach to introduce SMBG and weight, along with self-monitoring of diet and PA behaviors to help patients understand the relationship between their behaviors and outcomes.

To our knowledge, our study is the first to combine self-monitoring of diet, PA, and weight using a mobile app that are used in behavioral weight loss interventions along with a connected glucometer to help patients learn their behavioral patterns in association with their weight and blood glucose outcomes. Previous studies had used either connected glucometers along with access to a live certified diabetes educator coach [34] or personalized feedback messages based on connected glucometer results [35] for general T2DM patients, not specifically targeting overweight or obese T2DM patients from underserved communities.

Several limitations to this study should be acknowledged. First, the study sample was recruited from an underserved community in an urban setting, so the study findings may not be generalizable to underserved communities in rural areas. Second, the focus of this study was feasibility and acceptability; thus, the study did not have sufficient power to detect group differences. Third, we provided smartphones and Bluetooth-enabled glucometers to the participants because none of the study participants reported owning a smartphone; the adherence to self-monitoring may be different for those who previously owned a smartphone. Fourth, our measure on adherence to self-monitoring of PA depended on patient adherence to the recommended PA behaviors. Although this approach has been used in several other studies [29,36], it may underestimate the actual adherence to self-monitoring of PA. For example, our adherence to self-monitoring of PA was lower than adherence dietary self-monitoring, which could suggest that participants did not exercise at all on that particular day, they did not bother to enter 0 for exercise minutes, and instead, they left it blank. Future research should examine the difference between adherence to self-monitoring and adherence to the actual behavior separately. Fifth, our study only looked at the short term outcomes; maintaining long-term effect may be a different challenge that future studies should consider examining.

### Conclusions

Delivering a behavioral lifestyle intervention enhanced with multiple-behavior self-monitoring using smartphone apps and a connected Bluetooth glucometer in an underserved community is feasible and acceptable, and using mobile tools including smartphone apps and connected glucometers has the potential to increase patient adherence to self-monitoring of multiple behaviors and improve glycemic control among underserved populations. A full-scale randomized controlled trial is needed to confirm the findings of this feasibility trial.

## Appendix

Multimedia Appendix 1 CONSORT‐EHEALTH checklist (V 1.6.1).

## Abbreviations

BMI: body mass index
CONSORT: Consolidated Standards of Reporting Trials
CVD: cardiovascular disease
GLB: Group Lifestyle Balance
HbA _1c_: glycated hemoglobin
ITT: intention-to-treat
LOCF: last observation carried forward
AHEAD: Action for Health in Diabetes
mHealth: mobile health
PA: physical activity
PDA: personal digital assistant
PI: principal investigator
SMBG: self-monitoring of blood glucose
T2DM: type 2 diabetes mellitus
